# Shotgun metagenomics detects the human pegivirus complete genome in a pediatric patient with acute hepatitis of unknown etiology: a case report

**DOI:** 10.3389/fgene.2025.1653082

**Published:** 2025-09-11

**Authors:** Roberta Vazzana, Alessandra Mularoni, Claudia Vaiana, Andrea Cona, Giovanni Mulè, Caterina Amato, Giusy Ranucci, Pier Giulio Conaldi, Valentina Agnese, Nicola Cuscino, Alessia Gallo

**Affiliations:** ^1^ Department of Research, IRCCS-ISMETT (Istituto Mediterraneo per i Trapianti e Terapie ad alta specializzazione), Palermo, Italy; ^2^ Unit of Infectious Diseases, IRCCS-ISMETT (Istituto Mediterraneo per i Trapianti e Terapie ad alta specializzazione), Palermo, Italy; ^3^ Pediatric Department for the treatment and study of abdominal diseases and abdominal transplantation, IRCCS-ISMETT (Istituto Mediterraneo per i Trapianti e Terapie ad alta specializzazione), Palermo, Italy

**Keywords:** clinical metagenomics, hepatitis, viruses, infectious diseases, next-generation sequencing, case report

## Abstract

Human pegivirus (HPgV) is a positive-sense, single-strand RNA virus belonging to the Flaviviridae family. Although not conclusively linked to a specific disease, an increasing number of studies have recently reported an association between this virus and different human pathologies. In this study, we present a 6-month-old female infant admitted to the hospital for severe acute hepatitis. Her clinical history started with a one week of fever and diarrhea treated with paracetamol and amoxicillin–clavulanate for a total of 4 days. The persistence of the symptoms, high levels of transaminases, coagulopathy, increased lymphocytosis, and C-reactive protein (CRP) in the blood suggested an acute hepatitis episode. Serological and molecular biology tests for hepatotropic and non-hepatotropic viruses, including hepatitis B virus (HBV), hepatitis A virus (HAV), hepatitis C virus (HCV), hepatitis E virus (HEV), Epstein–Barr virus (EBV), cytomegalovirus (CMV), herpes simplex virus (HSV), enterovirus, and adenovirus, were negative. Metabolic and genetic alterations, deficiency of alpha-1 antitrypsin, and Wilson’s disease were ruled out following negative results. The child was thus treated with supportive therapy. Metagenomic next-generation sequencing (mNGS) performed to identify other possible infective agents undetected with the classical tests, showed the presence of the complete genome of human HPgV-1. This case provides further evidence supporting the hypothesis of the pathogenic role of HPgV-1 and warrants particular attention, especially in the pediatric population. Moreover, here we confirmed the diagnostic power of metagenomic-NGS in the detection of unusual pathogens.

## Introduction

Acute hepatitis is a clinical term used to define a complex condition that can lead to liver dysfunction. It is generally caused by an inflammatory state of the hepatic parenchyma or by injured hepatocytes. The clinical manifestation of acute hepatitis includes fever, jaundice, and altered liver function. This critical condition can be caused by both infectious and non-infectious events. The most common infectious cause of hepatitis is the viral infection by hepatotropic viruses, including hepatitis B virus (HBV), hepatitis C virus (HCV), and hepatitis A virus (HAV). Although it is rare, other non-hepatotropic viruses, such as Epstein–Barr virus (EBV), cytomegalovirus (CMV), herpes simplex virus (HSV), parvovirus B19, adenovirus, and enterovirus, can cause hepatitis. Drugs, toxins, autoimmune mechanisms, and immunologic or metabolic conditions are the most prevalent noninfectious causes of acute and chronic hepatitis (Stravitz and Lee, 2019).

Recently, the number of pediatric acute hepatitis cases of unknown etiology has increased around the world, leading to an intensified surveillance for the presence of specific infectious agents. Many studies reported indeed the co-presence of multiple viral infections as a possible explanation for several investigated outbreaks (Di Maio et al., 2024). Hence, the World Health Organization (WHO) considers the increased cases of acute hepatitis as a global public concern. Given this complex scenario, and despite the microbiological tests routinely performed, there is an urgent clinical need for unbiased approaches that may help identify infectious agents that may be responsible for diseases of unknown etiology.

Human pegivirus is a positive-sense single-stranded RNA (+ssRNA) virus that belongs to the Pegivirus genus of the Flaviviridae family. Although this virus, previously known as Hepatitis G virus (HGV) or GBV-C virus, was found in the past in patients with non-A-E hepatitis (Linnen et al., 1996; Simons et al., 1995; Heringl et al., 1996), further studies were not able to prove the direct correlations between the virus and the fulminant hepatitis cases. Interestingly, HPgV has been often found in patients in association with HCV (Ragupathy et al., 2025) and, in renal transplanted patients, a correlation has been reported between the co-infection of HGV/HCV and a higher percentage of acute rejection (Rostaing et al., 1999). More recently, an increasing number of case studies reported the exclusive presence of this virus associated with central nervous system infections (Carmona et al., 2023) and encephalitis (Tuddenham et al., 2020), while a cohort North American study showed that HPgV infection correlated with a higher risk of lymphoma development (Fama et al., 2018).

Metagenomic-NGS is one of the most sensitive techniques, especially in virus detection (Wu et al., 2024), that can provide a rapid diagnosis for multiple pathogens in a single experiment when combined with bioinformatic analyses.

A recent case of acute hepatitis of unknown etiology was experienced in our hospital. The patient tested negative for hepatitis B virus (HBV)/hepatitis C virus (HCV), and all the other analyses performed failed to identify a certain etiology. Using metagenomic-NGS on the plasma sample, we detected the presence of the complete genome of human HPgV-1 in the absence of other pathogens.

## Case description and diagnostic assessment

A 6-month-old female infant was admitted to the pediatric unit of a peripheral hospital after 1 week of fever and diarrhea. Following hospital admission, the patient received amoxicillin–clavulanate for a total of 4 days. Blood examinations showed worsening high levels of transaminases (AST/ALT up to 853/413 U/l), coagulopathy (INR 1.97), progressive increase of lymphocytosis, and moderate increase of CRP. The stool was negative for rotavirus and adenovirus. The alterations in the liver function tests observed suggested an acute hepatitis episode. Wilson’s disease was deemed unlikely due to normal ceruloplasmin levels. Alpha-1 antitrypsin deficiency, a genetic disorder that can cause liver disease, was also ruled out because alpha-1 antitrypsin levels were found within the normal range. Following a worsening of the clinical conditions, the child was transferred to our hospital for liver transplant evaluation.

At admission, vital parameters, including heart and respiratory rates, body temperature, blood pressure, and pulse oximetry, were within the normal range. Cardiac and chest auscultation were unremarkable. Neurological examination did not show any alteration. Her skin was well hydrated, and no jaundice or icteric sclera was observed. No pain or abdominal organomegaly was observed during the abdominal palpation. Complete abdominal ultrasound was generally normal but a diffuse and increased liver echogenicity without focal lesions was observed; moreover, a small right pleural effusion was shown. Blood examinations indicated stable hepatic cytolysis, hyperammonemia, and increased creatine phosphokinases (CPK). Paracetamol blood levels were within the normal range.

Metabolic and genetic hepatitis causes were investigated by using genetic screenings, biochemical assays, and ultrasound exams. Next-generation sequencing analysis did not show any genetic mutation associated with metabolic diseases; moreover, normal amino acids, galactose, acylcarnitine blood levels, and organic acids urinary levels were observed. Cardiac ultrasound and fundus oculi inspection did not show any alteration. Wilson’s disease and alpha-1 antitrypsin deficiency were excluded, considering that the values obtained were within the normal range. Autoimmune hepatitis associated with hemolytic anemia was excluded because haptoglobin, lactate dehydrogenase (LDH), and bilirubin was within the normal range, and direct and indirect Coombs tests were negative. Blood examinations did not fulfil diagnostic criteria for hemophagocytic syndrome.

Molecular biology and serological tests for hepatotropic and non-hepatotropic viruses were performed, and the patient tested negative for all of them. In particular, HCV DNA and HBV DNA were tested by using the Xpert HCV Viral Load and Xpert HBV Viral Load, respectively (Cepheid GeneXpert, Sunnyvale, California). Real-time PCR assays were performed for CMV, EBV, HSV1, HSV2, enterovirus, adenovirus, and HEV (ELITe MGB Kits, ELITech Group, Puteaux, France). Qualitative immunoassays for HAV detection were performed by using Liaison Anti-HAV and Liaison HAV IgM (Diasorin, Saluggia, Italy), while HHV-8 DNAemia was performed using the HHV-8 ELITe MGB Kit (ELITechGroup, Puteaux, France). VZV DNA, HHV-6 DNA, and parvovirus B19 DNA were also negative. Serological tests for *Toxoplasma* spp were negative.

**FIGURE 1:**
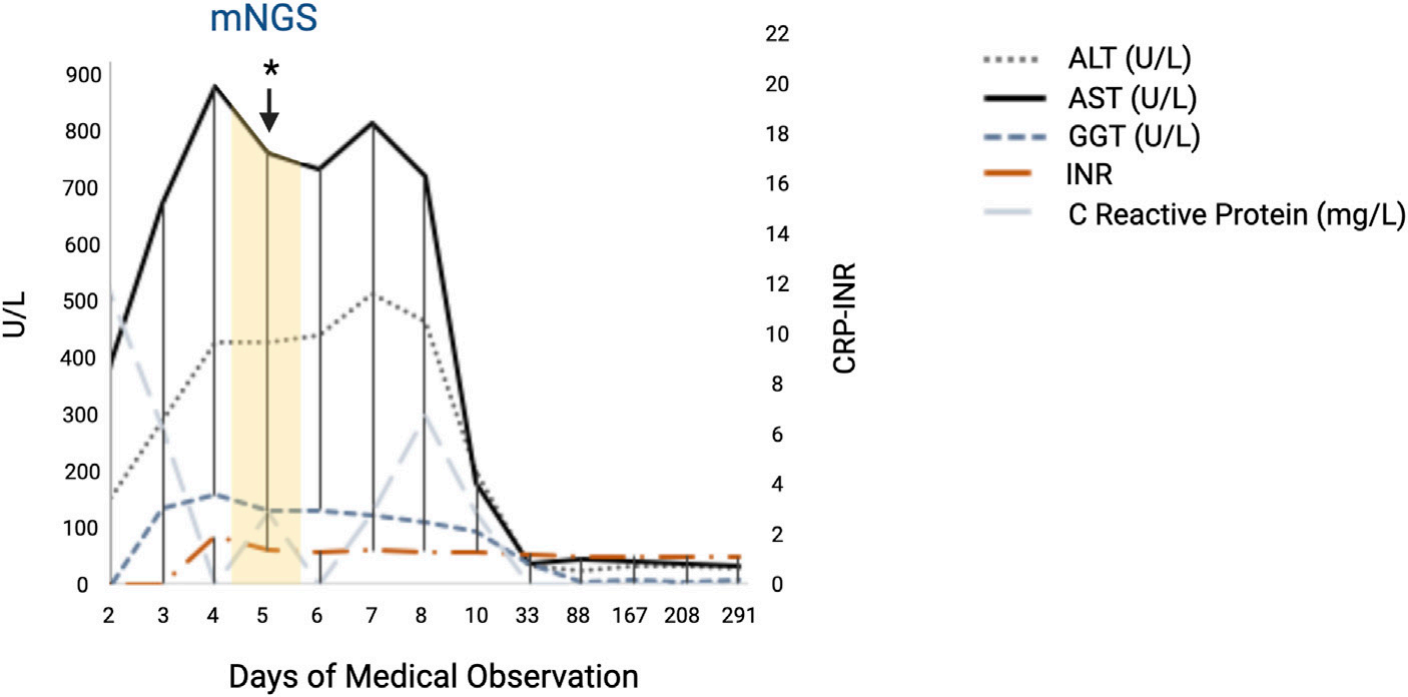
Main liver function test values of the patient showing the rapid increase in AST and ALT in the acute phase of the hepatitis. All the values reported represent the results collected in both hospitals, starting from day 2 of hospitalization and during the follow-up. Day 3 of medical observation represents the admission day in our hospital. The day on which the plasma sample was collected for clinical metagenomic analysis is shown in yellow. Abbreviations: ALT, alanine aminotransferase; AST, aspartate transaminase; GGT, gamma-glutamyl transferase; INR, international normalized ratio. This figure was created using BioRender (https://www.biorender.com/).

A diagnosis of acute hepatitis of unknown etiology was thus considered, and the child was treated with supportive therapy. In order to investigate other possible infectious causes, blood specimens were taken on the fifth day after hospitalization to perform metagenomic-NGS. On the tenth day of hospitalization, a progressive normalization of transaminases, INR, CPK, ammonium, and CRP levels was observed; moreover, vital parameters and the general condition of the patient were normal, thus the child was discharged with regular follow-up. At the following follow-up appointments, scheduled at 1 month, 3 months, 6 months, and 12 months, she was in good general condition and asymptomatic, with blood tests showing a normal hepatic function. The main liver function test values collected during the entire hospitalization period are shown in Figure 1, and the remaining laboratory test values are shown in the Supplementary Material.

## HPgV identification by metagenomic-NGS and phylogenetic analysis

Metagenomic-NGS (mNGS) was performed from a plasma sample collected on the fifth day of hospitalization. Interestingly, the complete genome sequence of human pegivirus 1, with a genome size of 9,187 base pairs, was found.

In brief, viral nucleic acids were extracted by using the DSP Virus Spin Kit (Qiagen, Hilden, Germany), and a modified sequence-independent single-primer amplification (SISPA) method was used to enrich for the viral genomes (Lorusso et al., 2020). The library was prepared using an Illumina DNA Prep kit and Illumina Nextera DNA CD Indexes, according to the manufacturer’s protocol. Shotgun metagenomic-NGS was then performed using the Illumina NextSeq™ 550 platform (San Diego, CA, USA). Sequencing data were analyzed using MetaWRAP (Uritskiy et al., 2018). Contigs and reads were then used for sequence similarity search on the NCBI database.

**FIGURE 2:**
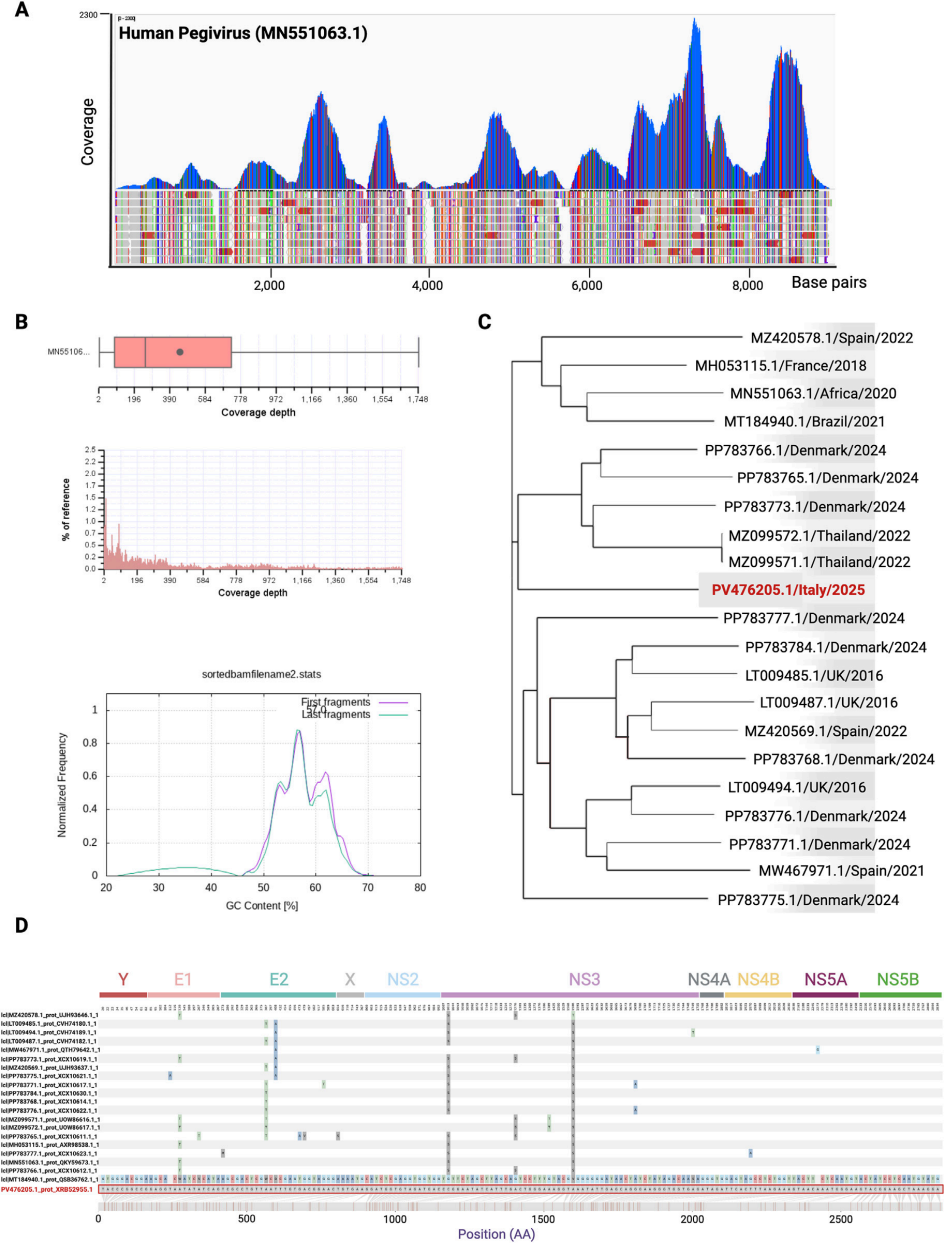
Coverage plot, distribution, and phylogenetic analysis of the HPgV-1 detected in the patient with acute hepatitis. **(A)** Report of the MetaWRAP analysis created using IGV software (Robinson et al., 2011). Nucleotide position is shown along the MN551063.1 HPgV isolate MAU-23 reference genome. **(B)** Box and whisker summary and histogram showing the coverage mean and distribution of the alignment. GC content % normalized on the frequency is shown in the lower panel. Reads mapping statistics and quality from BAM files were calculated by using the RSeQC module bam_stat.py (9), samtools-stats, and ploy-bam stat (10). **(C)** Phylogenetic tree of HPgV sequence reads obtained in this study (GenBank accession number PV476205.1, indicated in bold dark red) and the set of 20 genomes used for the phylogenetic analysis with the corresponding NCBI accession number, year, and country of origin. The tree was generated using Harvest software (Treangen et al., 2014). **(D)** Amino acid sequence visualization alignment of the HPgV-1 strain identified in this study and the top 20 most related strains. The figure was created by using the Snipit tool for amino acid visualization (GitHub repository, https://github.com/aineniamh/snipit, O'Toole et al. (2024)). The HPgV-1 strain identified in this study is highlighted in red. The positions of the single proteins were predicted by alignment and homology to genes previously characterized in other pegiviruses (Kapoor et al., 2015). This figure was created using BioRender (https://www.biorender.com/).

A total of 27,239 reads were detected from the HPgV virus. These reads were analyzed and remapped against the closest genome sequence for percentage of identity according to the BLASTn database, MN551063.1 human pegivirus isolate MAU-23. Compared to this reference, the genome identified showed 91% identity, with a query coverage of 98% (Figure 2A). With a coverage mean of 447.20×, the genome showed an average percentage of GC content (GC%) of 57.04% (Figure 2B). The genome possessed a single ORF of 8,529 nt from position 499 to position 9,027, codifying for a 2,842 aa polyprotein. The phylogenetic tree, generated by comparing the ORF encoding the identified polyprotein together with the 20 reference sequences yielding the highest BLASTn alignment scores, showed that the genome formed an independent branch. This branch clusters with five different strains of human pegivirus genotype 1 that were previously reported in Thailand (2022) and Denmark (2024) (Figure 2C). The novel sequence identified here has been assigned GenBank accession number PV476205.1. The genome sequence deposited on GenBank represents a consensus sequence built with a high coverage depth (447.20×) and represents the predominant genome sequence of HPgV-1 virus found in the patient’s sample. Minor variants or mutations were not annotated.

Moreover, we performed an ORF multiple alignment by comparing the amino acid sequence newly identified in this study with the top 20 most related strains. The multiple alignment showed two amino acid changes at positions 1011 and 1586 that are highly conserved among all the strains compared. These aa mutations (Ser1011-Cys and Ser1586-Ala) occur within the NS3 region (non-structural protein). Other aa mutations occur within the E1 and E2 regions (structural proteins) but in less conserved residues, for example, Thr130-Ala, Thr387-Ala, and Ala408-Thr, as shown in Figure 2D.

## Discussion

Although the number of pediatric hepatitis cases is increasing worldwide, unfortunately, more than 30% of the cases were of unknown origin (Rodriguez-Frias et al., 2023) (WHO: Acute Hepatitis of Unknown Etiology the United Kingdom of Great Britain and Northern Ireland; WHO, Geneva, Switzerland: 2022 https://www.who.int/emergencies/disease-outbreak-news/item/2022-DON368). This led recently to improved surveillance among clinicians. Viruses are considered among the most common etiological agents. Acute hepatic dysfunction in the pediatric population is often associated with hepatitis viruses (A–E), but non-hepatitis viruses (like CMV, EBV, HHV-6, adenovirus, and parvovirus B19) can also trigger liver failure (Gallegos-Orozco and Rakela-Brodner, 2010). Recently, the increased sensitivity and specificity of the detection methods used in the infectious disease field, together with the use of unbiased approaches, have also allowed the detection of rarer viruses that may have an impact on patient outcomes.

In this study, we found no evidence of the presence of any hepatotropic or non-hepatotropic virus that could be associated with acute liver failure, including HBV, HAV, HCV, HEV, EBV, CMV, HSV, VZV, HHV-6, HHV-8, parvovirus B19, enterovirus, adenovirus, and rotavirus. Other non-infective possible causes were considered during the diagnostic assessment of the patient, including drug-induced liver damage, autoimmune hepatitis, and metabolic and genetic diseases, but they were all excluded following the negative results obtained with the screenings performed.

Shotgun metagenomics was thus used in the attempt to investigate, with an unbiased approach, the presence of other potential infectious agents not detected with the classical diagnostic tests previously performed. Remarkably, the presence of the complete genome sequence of HPgV-1 was detected, without the concomitant presence of any other hepatotropic virus, especially HCV, whose co-presence has been reported previously in many patients with acute hepatitis (Ragupathy et al., 2025). Moreover, the phylogenetic analysis showed that the HPgV-1 strain PV476205.1/2025 belongs to a well-defined singular clade and shows its genetic association with previous HPgV strains recently identified in Thailand and Denmark.

Human pegivirus, initially referred to as hepatitis G virus or GB virus C, was first identified in the plasma of a patient with chronic hepatitis (Linnen et al., 1996; Simons et al., 1995). This virus shows genomic similarities with the hepatitis C virus, and previous studies revealed a correlation between fulminant hepatic failure and the presence of GB virus C (Heringl et al., 1996). Considering these old observations, for many years the scientific community tried to understand whether this virus was associated with the hepatitis cases, and further studies were not able to demonstrate a direct correlation between the virus and the hepatitis cases.

Currently, HPgV has not been conclusively linked to a specific disease, notwithstanding that many studies have lately reported an association between its presence and different human pathologies (Carmona et al., 2023; Tuddenham et al., 2020; Fama et al., 2018; Scheibe et al., 2025), including an increased risk of lymphoma and encephalitis. Indeed, recent findings show that this virus can be considered primarily lymphotropic (Kapoor et al., 2015), but its presence was also detected with a high viral load in the central nervous system of patients with encephalitis of unknown etiology (Bukowska-Osko et al., 2018; Fridholm et al., 2016). The use of viral metagenomics recently allowed the detection of HPgV in the cerebrospinal fluid of patients with central nervous system (CNS) infections of unknown etiology (Carmona et al., 2023) and in immunocompromised patients with myelitis and neuritis (Fridholm et al., 2016).

Notwithstanding that the detection of HPgV-1 virus alone does not prove its etiological role, the presence of the complete genome of the virus in the plasma sample of the infant patient, without the presence of other pathogens, represents an important aspect to be taken into consideration and warrants particular attention in the pediatric population.

Moreover, the efficiency of the metagenomic methodology in identifying unknown pathogens remains noteworthy. The use of metagenomic-NGS in the clinical context is recently becoming more established, thanks to the high sensitivity associated with the technique, the possibility to detect multiple pathogens in a single run, and the broad applicability across various biological specimens. In a multicenter study involving both pediatric and adult patients with infectious encephalitis and meningitis, metagenomic next-generation sequencing ameliorated diagnostic yield, providing clinically relevant information to guide the management of neurological infections (Wilson et al., 2019).

In conclusion, this study emphasizes the clinical importance of the use of metagenomic next-generation sequencing (mNGS) for the differential diagnosis of pediatric hepatitis cases that have recently increased worldwide. The use of this unbiased approach can allow the detection of potential infectious agents, especially in cases of unknown etiology, thus supporting clinicians during the diagnostic assessment.

## Data Availability

The viral genome sequence has been deposited in GenBank under the accession number PV476205.1 (https://www.ncbi.nlm.nih.gov/nuccore/PV476205.1/). Data supporting the results are provided within the Supplementary Material file. The raw Illumina sequencing mNGS data (with the removal of human reads) supporting the conclusion of this case report are available in the NCBI SRA database with the accession number: PRJNA1294276. The hyperlink to the database is the following: https://www.ncbi.nlm.nih.gov/bioproject/1294276.

## Abbreviations

CMV, cytomegalovirus; CNS, central nervous system; CRP, C-reactive protein; EBV, Epstein–Barr virus; HAV, hepatitis A virus; HBV, hepatitis B virus; HCV, hepatitis C virus; HEV, hepatitis E virus; HHV-6, human herpesvirus 6; HHV-8, human herpesvirus 8; HPgV, human pegivirus; HSV, herpes simplex virus; NGS, next-generation sequencing; VZV, varicella zoster virus.
